# The alterations of degree centrality in the frontal lobe of patients with panic disorder

**DOI:** 10.7150/ijms.65367

**Published:** 2022-01-01

**Authors:** Yongbao Liu, Chien-Han Lai

**Affiliations:** 1 Department of Imaging, The First People's Hospital of LianYun Gang, Lianyungang City, Jiangsu Province, 222000, China; 2 Institute of Biophotonics, National Yang-Ming University, Taipei, Taiwan; 3 PhD Psychiatry & Neuroscience Clinic, Taoyuan, Taiwan

**Keywords:** panic disorder, degree centrality, superior frontal gyrus, inferior frontal gyrus, alterations

## Abstract

**Objective:** The brain network in panic disorder (PD) is still an intriguing issue for research. In this study, we hoped to investigate the role of DC (degree centrality) for the pathophysiology of PD, especially for the fear network.

**Methods:** We enrolled 60 patients with PD and 60 controls in the current study. The gender and age were matched for two groups. All participants received the resting-state functional magnetic resonance imaging to survey the baseline brain activity. Then the DC values of all participants were using REST toolbox. We also compared the DC values between PD and controls. The statistical threshold was set as FDR (false discovery rate) < 0.05.

**Results:** The DC values were significantly lower in the right superior frontal gyrus of PD patients compared to controls (FDR < 0.05). In addition, a negative correlation between the DC values and panic severity was observed in the right superior frontal gyrus and left inferior frontal gyrus. However, there was no significant association between the DC values and illness duration.

**Conclusion:** The DC seemed significantly altered in the frontal lobe of PD patients. The role of the frontal lobe might be more emphasized in the pathophysiology research for PD.

## Introduction

Panic disorder (PD) is characterized of autonomic nervous system dysfunction, such as dizziness, chest tightness, palpitations and other somatic complaints. The neuroanatomy hypothesis of PD basically focused on the fear network hypothesis, including the frontal lobe, thalamus, amygdala, hippocampus, hypothalamus and brainstem 1. In recent years, another review article of PD hypothesis suggested the potential extension of fear circuit areas into more regions, such as the anterior cingulate and insula. 2.

The default brain activity has been applied in the neuroscience research for recent one decade. It can be evaluated by the resting-state functional magnetic resonance imaging (Rs-FMRI) technique. There are many parameters for the measurement of default brain activity. Among the parameters, the degree centrality (DC), a measurement of centrality, connectedness and influences of a node within a network. The node's prominence profile can reveal the network dynamics and node centrality 3. For the viewpoint of Rs-FMRI, the DC can provide the information for the number of instantaneous functional connections between a region and the rest of the brain within the entire brain connectome. Therefore the DC values can reveal the node influences for entire brain areas 4. It has been applied in the pathophysiology study for many kinds of illness, such as depression 5-7, schizophrenia 8, bipolar disorder 9 and end-stage renal disease 10. The application of DC study in anxiety pathophysiology is rare 11. For the PD, the role of DC has not been addressed much.

In the fear network model for PD, the crucial role of frontal cortex and limbic regions has been proved in the several studies. The blood perfusions were altered in the frontal and limbic regions, such as hippocampus, in the patients with PD 12. The benzodiazepine receptor uptake was reduced in the frontal cortex of PD patients 13. PD patients also showed increased brain activities in the left inferior frontal cortex and hippocampus when exposing to emotion-related memory 14. Our previous report of remitted PD patients also demonstrated the changes in the regional homogeneity of frontal and temporal regions after antidepressant treatment 15. These reports supported the importance of frontal cortex and limbic regions in the PD pathophysiology.

In current study, we wanted to utilize the DC to survey the altered pattern in the patients with PD. From the fear network hypothesis and the literature mentioned above, we hypothesized that the alterations of DC would be observed in the frontal cortex and limbic regions. The possible correlations between panic severity, duration of illness and DC values might be also found the frontal cortex and limbic regions.

## Methods

All patients and healthy subjects signed the informed consent that was approved by the three Institutional Review Boards (Taipei Tzu Chi General Hospital, Cheng Hsin General Hospital and National Yang-Ming University) according to the institute where they were recruited. Informed consent was obtained from all of the individual participants included in the study. None of PD patients had comorbid depression. The PD group was enrolled according to the following criteria: (1) first-episode patients with a pure PD diagnosis (DSM-IV criteria) (2) The severity of PD was at least moderate: Clinician Global Impression of Severity > 4, Quick Inventory for Depressive Symptoms-Self Rating 16-item version (QIDS-SR16) < 9, Hamilton Rating Scales for Depression (HDRS) score < 7, Hamilton Rating Scales for Anxiety (HARS) score > 22, Panic Disorder Symptom Severity Scale (PDSS) > 15, panic attacks of full blown symptom > 4 times within previous 4 weeks before the baseline visit. (3) no co-morbid psychiatric illnesses or medical illnesses; (4) no previous cognitive behavioural therapy or other psychotherapies; (5) medication-naïve; (6) no abuse of or dependence on alcohol or other substances; and (7) no past history of claustrophobia or discomfort while receiving MR scanning. The healthy controls had no psychiatric illnesses or significant medical illnesses. The patients were enrolled at two general hospitals. The controls were enrolled from 2 general hospitals and one university. At the time of the MR imaging, none of the participants in the control group received psychotropic treatment. Handedness was determined by using the Edinburgh Inventory of Handedness 16. We screened 101 patients with PD and 67 controls. Finally, we enrolled 60 patients with PD and 60 controls according to the above selection criteria.

### Structural MRI (magnetic resonance imaging) data acquisition

The structural MRI T1 imaging scans of brain were obtained with 3T Siemens version scanner housed at MRI Center, National Yang Ming University. Scans with three-dimensional fast spoiled gradient-echo recovery (3D-FSPGR) T1W1 (TR 25.30ms; TE 3.03ms; slice thickness = 1mm (no gap); 192 slices; matrix = 224x256; field of view: 256mm; number of excitation=1) were performed.

### Rs-FMRI data acquisition and pulse sequence

Echo planar imaging (EPI) sequence were acquired in 20 axial slices (TR=2000ms, TE=40ms, flip angle=90°, field of view=24cm; 5mm thickness and 1 mm gap; the sequence duration was 300 seconds for each subject, 150 time points were acquired, voxel dimension: 64x64x20) at baseline visit (3T Siemens scanner housed at MR center of National Yang Ming University) in patients and controls. All the patients and controls were requested to close their eyes with relaxing manner and not sleepy while scanning. The participating subjects were instructed to move as little as possible and stay fully awake while scanning. All these patients and controls reported that they could be fully awake while MRI scanning.

### Rs-FMRI preprocessing and DC analysis

EPI data was first preprocessed by DPARSF (Data Processing Assistant and Resting-State FMRI, version 2.2; State Key Laboratory of Cognitive Neuroscience and Learning, Beijing Normal University, Beijing, China.) 17 working with the statistical parametric mapping 8 (SPM8) on the Matlab platform, which included the removal of first 10 time points due to the consideration for instability of initial MRI signal and patients' difficult to adapt at first about MRI acquisition circumstance, slice timing with 20^th^ slice as reference slice, realignment, normalization to standard MNI spaces by using EPI templates and re-sampling with 3 x 3 x 3 mm3, smoothing by Full Width at Half Maximum (FWHM) 4x4x4 kernel, to detrend and filter data with residual signals within 0.01-0.08 Hz to discard the bias from high-frequency physiological noise and low-frequency drift. As all subjects' head movements were less than 0.5 mm in translation and 1 degree in rotation by obtaining the motion time courses of all subjects, no subject was excluded due to no excessive motions were observed. The realignment estimates were also used for reducing motion contamination for the data 18. A study-specific MNI template was derived from segmented transformed images consisting of gray matter, white matter and cerebrospinal fluid into the MNI space. The filtered RFMRI data was registered (nonlinear elastric registration) to this symmetric study-specific MNI template, which was created by voxel-based morphometry of T1 images. The estimated motion parameters were obtained for each subject regressed on each voxel. Non-gray matter nuisances, such as the white matter and CSF signals, were regressed to reduce the motion artifacts 18. Several sources of spurious covariates were removed except global signals due to the controversy about removing the global signal in the resting-state functional connectivity data 19, 20. The global signal regression method combined with within-subject and censoring-based artifact removal strategy 21 were also used for reducing motion artifacts. The individual-level covariates of motion included Friston-24 parameter model (6 head motion parameters, 6 head motion parameters one time point before, and the 12 corresponding squared items) and group-level covariates of motion included framewise displacement motion regression model 22. We also implemented some denoising procedure that produces incompletely denoised time series (e.g., mean white matter signal regression), and then to use partial correlations rather than full correlations to study relationships among the time series. The final output data after DPARSF preprocessing was then processed by REST (Resting State FMRI Data Analysis Toolkit, version 1.4; State Key Laboratory of Cognitive Neuroscience and Learning, Beijing Normal University, Beijing, China.) 23 and DC data was produced subsequently.

The DC values of all subjects were produced by the REST toolbox. The whole-brain functional connectivity matrix would be produced using Pearson's correlation coefficients between the time series of all pairs of gray matter voxels. This computation followed the gray matter mask set by the previous article 24. The Fisher's r-to-z transformation was used to derive the Z-score matrix and improve normality. The regional functional connectivity strength was calculated as the sum of all the connections (Z-values) between voxels. The analysis was conservatively restricted to positive correlations above a threshold of r = 0.2. , which can eliminate the weak correlation due to signal noise. We identified the voxels with higher regional functional connectivity strength values, which usually indicate the centrality within the specific network of brain.

### Statistical analysis

The DC map was used for the following independent two sample t test analysis function implemented in the REST toolbox. The subsequent group comparison analysis between patients and controls was performed (second-level random effects model, independent two sample t test) under the following statistical criteria [false discovery rate (FDR) corrected p < 0.005, cluster > 10 voxels, T threshold: 5.1847, surface connected theory]. The above statistical analysis also used age and gender as covariates to exclude possible influences from these parameters. In addition, to clarify the relationship between clinical severity of panic symptoms and DC values, a voxel-wise Pearson's correlation was performed. For each subject, the Pearson correlation coefficient between each voxel's residual time series and that of its symmetrical inter-hemispheric counterpart was performed. The correlation values were then Fisher z-transformed to improve normality.

## Results

### Demographic data

There were no significant differences in age, gender, HDRS scores between the patient and control groups. However, significant differences were observed in the HARS scores and PDSS scores between patients and controls. All participants of the two groups were almost right-handed (SPSS version 16).

### The differences in the DC values between patients and controls

**Figure 1:**
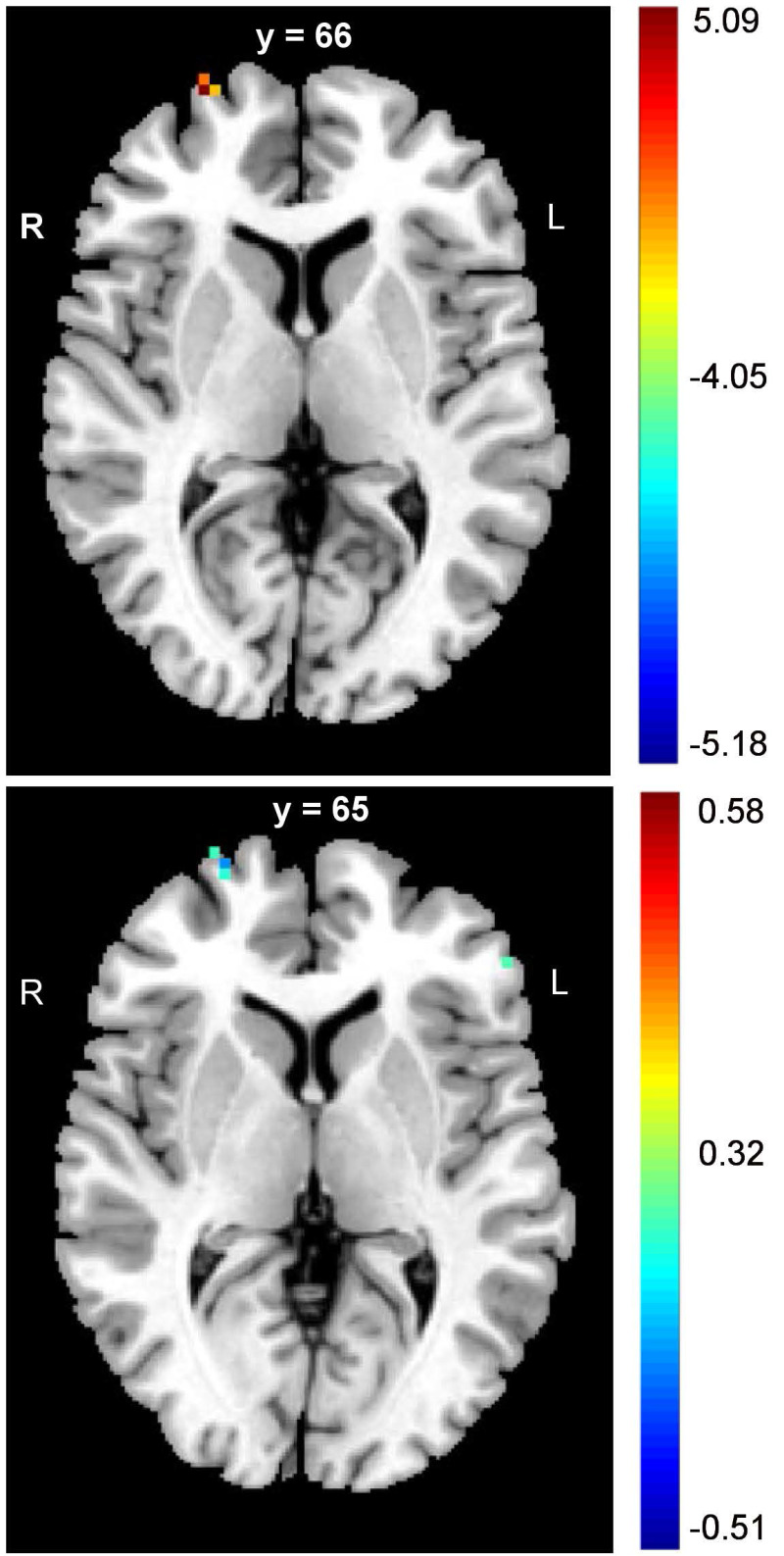
** The alterations of frontal cortex in DC values of PD patients.** The PD patients had lower values of DC in SFG when compared to controls (upper panel figure). In addition, the negative correlations between panic severity and DC values were observed in the right SFG and left IFG (lower panel figure). (MNI: Montreal Neurological Institute; SFG: superior frontal gyrus; IFG: inferior frontal gyrus)

The PD patients had significantly lower DC values in the right SFG (FDR corrected p < 0.05) (Table 2, upper panel of Figure 1). In addition, negative correlations between the DC values and panic severity were observed in the right SFG and left inferior frontal gyrus (IFG) (Table 2, lower panel of Figure 1). However, there was no significant association between the DC values and illness duration.

## Discussion

We found significant alterations in the DC values of frontal cortex, such as the right SFG in current study. However, the absence of DC alterations in the limbic system was incompatible with our hypothesis. In addition, we found significantly negative correlation between DC values and panic severity in the right SFG and left IFG. Such a correlation was not found between illness duration and DC values.

The alteration of DC in the frontal lobe, such as the right SFG, was the major finding in current study. In the panic-provoking model, it indicated that the activation of right SFG might play a role in the dysregulation of fear network model in PD 25. Another finding is the negative correlation of panic severity with the DC values in the left IFG and right SFG. This finding is also compatible with the antidepressant-related modulations in the metabolic and regional homogeneity in the frontal regions of PD patients 15, 26. In addition, the stimulation of SFG also can modulate inhibitory control for motor response, which might be important for the panic responses using the motor muscles 27. Our study result of left SFG also corresponded to the inhibitory control hypothesis of PD 27. The SFG can regulate goal-directed top-down process and is a part of dorsal attention system 28. According to the study of resting-state functional connectivity, the SFG is connected with the thalamus and other limbic regions, which are also components of fear circuitry of PD 29. However, this theory might need further resting-state function connectivity to support our results of DC alterations in SFG. In the previous model of short-term treatment of paroxetine for PD, the post-treated patients showed significant changes of glucose metabolisms in frontal regions 26. It supported the crucial role of frontal region in the pathophysiology and clinical responses in PD. From the above literature, SFG might interact with other limbic regions within fear circuitry for inhibition of panic responses, which also suggest the important role of SFG in etiology of PD. However, current study results found no significant alterations of DC values in the limbic system. It can be explained as that maybe DC values might not be such sensitive as a biomarker for the alterations in limbic system of PD patients, even without abundant study findings of DC in PD can support this explanation. Maybe researchers can perform more DC studies in PD and focus on the limbic system in the future. If we combine the above literature with the findings in current study, it suggests that the SFG might play the role for the trait-dependent biomarker for DC, which may constitute the blueprint of network centrality and stability of PD.

The negative relationship between DC values and panic severity can be an interesting point for understanding the role of DC in the pathophysiology of PD. From the structural point, it indicated the importance of IFG in the pathophysiology of PD and the relation to the severity of PD 30-32. The panic-specific pictures will also induce greater activation in the IFG and limbic regions in PD patients 33. The later study also replicated the findings of IFG in the responses to the panic-related pictures in PD patients 34. The IFG-related inhibitory control and impacts to the fear network circuitry might be also associated with the panic response 35. The right-left asymmetry and decreased activities of cerebral blood flow in the IFG was also found during panic attacks 12, 36, which might also explain the correlation of DC values with the panic severity. The impairments of cognitive function 37 and hypofrontality was also found in the left IFG of PD patients 38. The alteration of functional MRI signals in the IFG was also observed during the processing of panic-related stimulus 39 and the recognition of negative words 40. The functional MRI study of cognitive behavioral therapy also showed that the IFG might be cognitive processing core for the improvement in panic syndrome. In contrast, the typical fear network was responsible for emotional processing in the remission of panic symptoms 41. Therefore from our study results, the correlation between DC values and panic severity may suggest that the role of DC values in the pathophysiology of PD.

Our study is the first study of DC values in PD patients. We also found the important role of frontal regions, such as SFG and IFG, in the DC pathophysiology model for PD. Our study results can advance our understandings about the formation of PD from the viewpoint of DC, especially for the frontal region within the fear network model. The SFG and IFG are the crucial regions for cognitive function. The study of antidepressant treatment showed the treatment response accompanied with the changes in glucose metabolism in the IFG and SFG^42^, which might play a role in the explanation of current study results. Another study also demonstrated the involvement of IFG in the response of antidepressant therapy in PD 26. PD patients might also have a prolonged activation in the left IFG during extinction recall 43. The negative correlation of DC values with panic severity in the IFG might be associated with this kind of involvement. The functional MRI study of panic-related words and emotional Stroop task showed that the processing function of the attention and emotion would be associated with alterations of activities in the IFG of PD patients 39. The emotional word recognition task also provoked the heightened signals in the IFG 40. The functional MRI study of cognitive behavioral therapy in PD suggested that the cerebral correlates of IFG would interact with the limbic regions in the improvements of PD symptoms 41, which corresponded to the decoupling sign between IFG and limbic regions of another study 44. The transcranial magnetic stimulation study also replicated the importance of IFG in the treatment response in PD 45. The anxiety-related trait and dimension also point to the IFG 46. From the above literature, the SFG and IFG may be the core region for the top-down mechanism of pathophysiology in PD 47. Our findings in current study might support the crucial role of SFG and IFG in the pathophysiology of PD. The relationship with the panic severity might suggest the potential biomarker for PD. However, the further study with a well-designed architecture can help us confirm the hypothesis.

First, the cross-sectional design is a major weakness. A future longitudinal study would help use confirm the importance of DC in the IFG and SFG of PD patients. Second, the lack of cognitive function and neuropsychological data in current sample might limit explanation of cognitive core of IFG and SFG and the relationship with DC values for PD. Third, functional connectome measures the functional connectivity according to the signals of hemodynamics. The alterations of DC values are probably related to the hemodynamic changes. However, it is still unknown whether the method of DC can be used to detect early neuronal changes, or monitor disease progression in PD. Fourth, a task-oriented functional MRI study could complete the viewpoint due to Rs-FMRI characteristics of the functional connectome results. A future study combined with task-oriented functional MRI method can help us confirm the findings of DC values in current study.

## Conclusion

From current study results, the crucial role of frontal lobe in PD can be proved in the study of DC alterations. The SFG might be an important biomarker for DC. In addition, the negative correlation of DC values with panic severity in the IFG might be another crucial part for the DC alterations in PD patients.

## Figures and Tables

**Table 1:** Demographic data of participating patients and controls

	Patients (N=60)	Controls (N=60)				Sig p (2-tailed), Z df=118
Age, mean (SD), years old	43.28 (9.66)	40.71 (10.38)			0.109, -1.61	
Gender (number)	F(30), M(30)	F(30), M(30)			0.715	
Duration of illness, mean (SD), months	5.46 (2.28)	0 (0)			N/A	
Educational years, mean (SD)	15.96 (1.05)	16.23 (0.83)			0.102, -1.636	
Handedness	R (58), L (2)		R (58), L (2)			0.152
HDRS, mean (SD)	1.15 (0.86)		0.96 (0.89)			0.21, -1.603
HARS, mean (SD)	23.21 (2.66)			1.22 (0.91)		<0.001, -9.571
PDSS, mean (SD)	21.40 (1.83)			N/A		N/A
						

N: number; SD: standard deviation; F: female, M: male; HDRS: Hamilton rating scales for depression; HARS: Hamilton rating scales for anxiety; PDSS: panic disorder severity scale; N/A: not applicable; Sig p (significance of p-value) was from Mann-Whitney U test for nonparametric independent 2-sample t-test; df: degree of freedom.

**Table 2:** The DC alterations and negative correlations between DC and panic severity in the frontal lobe of PD patients

Region	MNI coordinate x y z	Cluster voxels	Statistical significance
Right SFG	27, 66, 6	47	FDR corrected p < 0.05, peak intensity: 5.0685
Right SFG (correlation)	27, 65, 4	50	FDR corrected p < 0.05, r: -0.51
Left IFG (correlation)	-53, 37, 4	41	FDR corrected p < 0.05, r: -0.42
